# Supplementary material for: Association of Alcohol Types, Coffee, and Tea Intake with Risk of Dementia: Prospective Cohort Study of UK Biobank Participants
Source: Brain Sci. 2022 Mar 8;12(3):360. doi: 10.3390/brainsci12030360 (PMC8946788; doi:10.3390/brainsci12030360)
Supplement: Supplementary file 1 [file brainsci-12-00360-s001.zip › brainsci-1608284-supplementary.pdf]

Online Supplementary Material

Association of alcohol types, coffee, and tea intake with risk of dementia: prospective cohort study of UK Biobank participants

Schaefer SM et al.

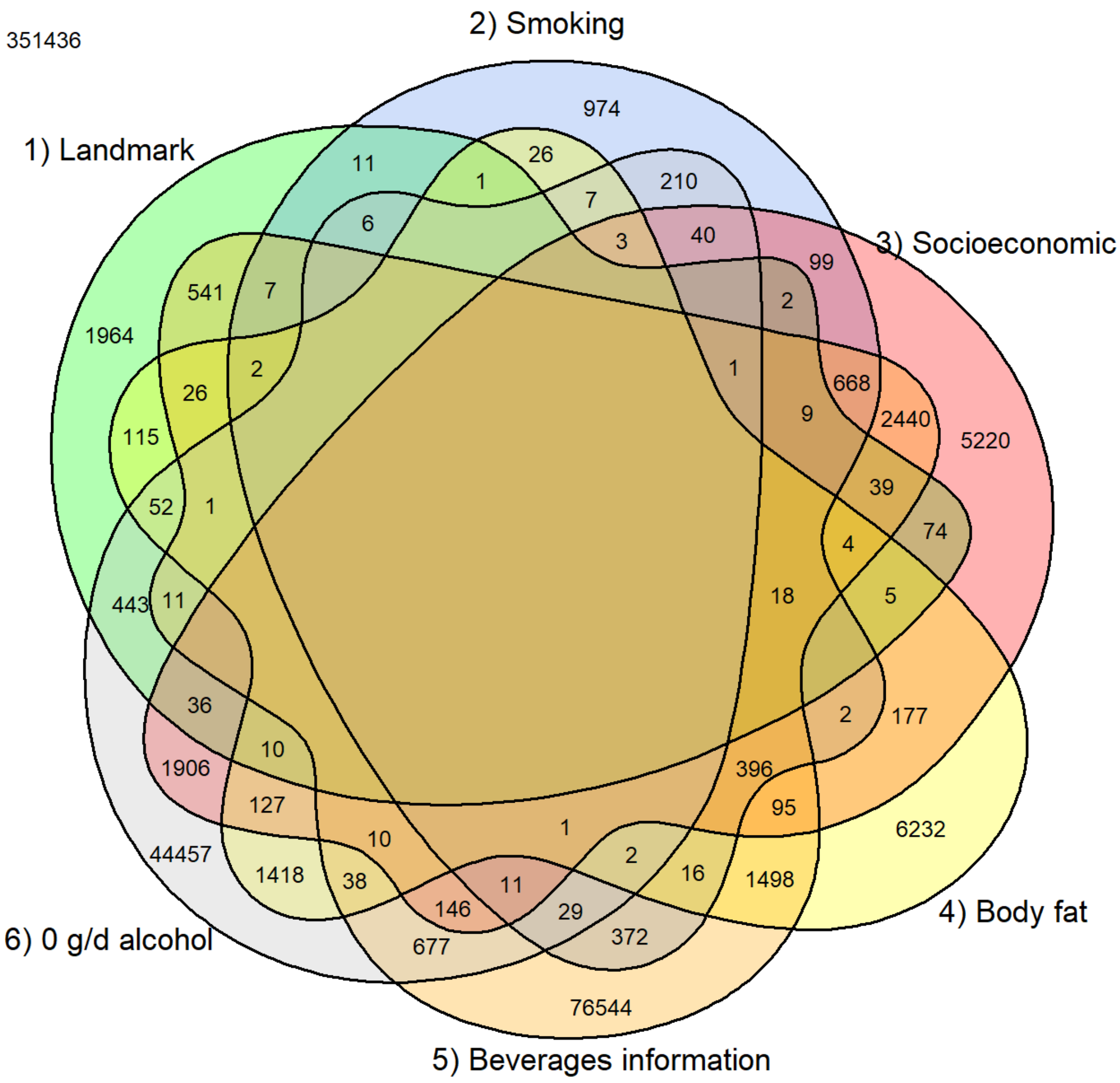

Figure S1. Venn diagram depicting number of participants excluded by six exclusion criteria (primary cohort). The number of participants for which exclusion criteria apply is depicted as colored shape and labelled as follows: Participants with 1) pre-existing dementia at baseline and incident dementia within 2 years after baseline in green, 2) missing smoking status in blue, 3) missing socioeconomic factors (i.e., annual household income (AHI), ethnicity, highest qualification, and/or overall health rating (OHR)) in red, 4) missing percentage body fat in yellow, 5) either missing information on beverage intake or being in the upper 0.1 % of alcohol, coffee, or tea consumption in orange, 6) present alcohol intake of 0 g alcohol/d in grey.

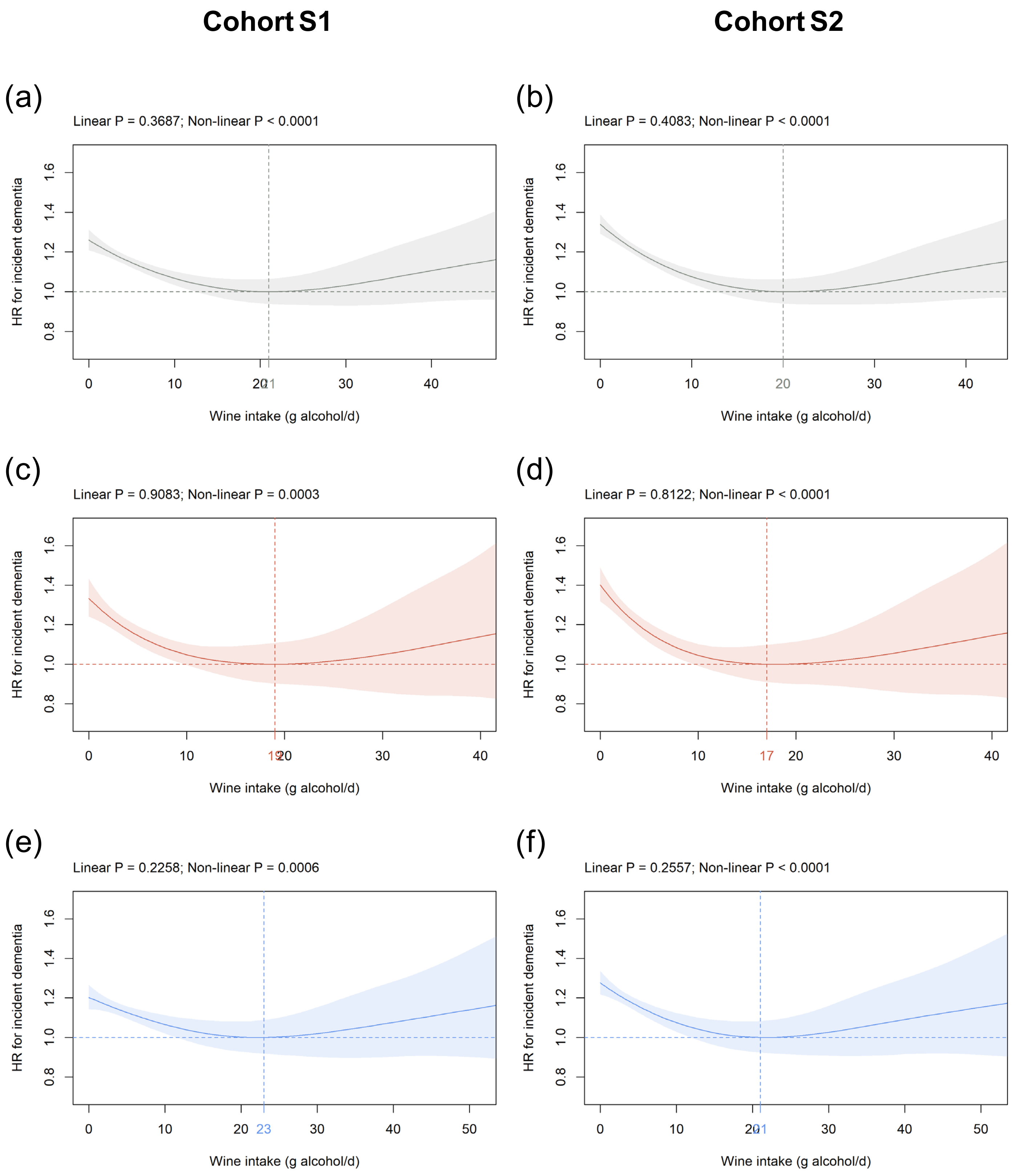

Figure S2. Association of wine intake (g alcohol/d) in (a), (b) the total cohort, (c), (d) females, and (e), (f) males with dementia risk in (a), (c), (e) cohort S1 and (b), (d), (f) cohort S2. Data are adjusted for sex (all participants only), age, AHI, ethnicity, highest qualification, OHR, PA, percentage body fat, and smoking status. Additionally, wine, non-wine, coffee, and tea intake are mutually adjusted (e.g., wine intake is additionally adjusted for non-wine, coffee, and tea intake) as summarized in the Materials and Methods section. Covariates not fulfilling the proportional hazard assumption are stratified. The nadir is indicated in grey (total cohort), red (female), and blue (male). AHI, Annual household income; HR, Hazard ratio; OHR, Overall health rating; PA, Physical activity.

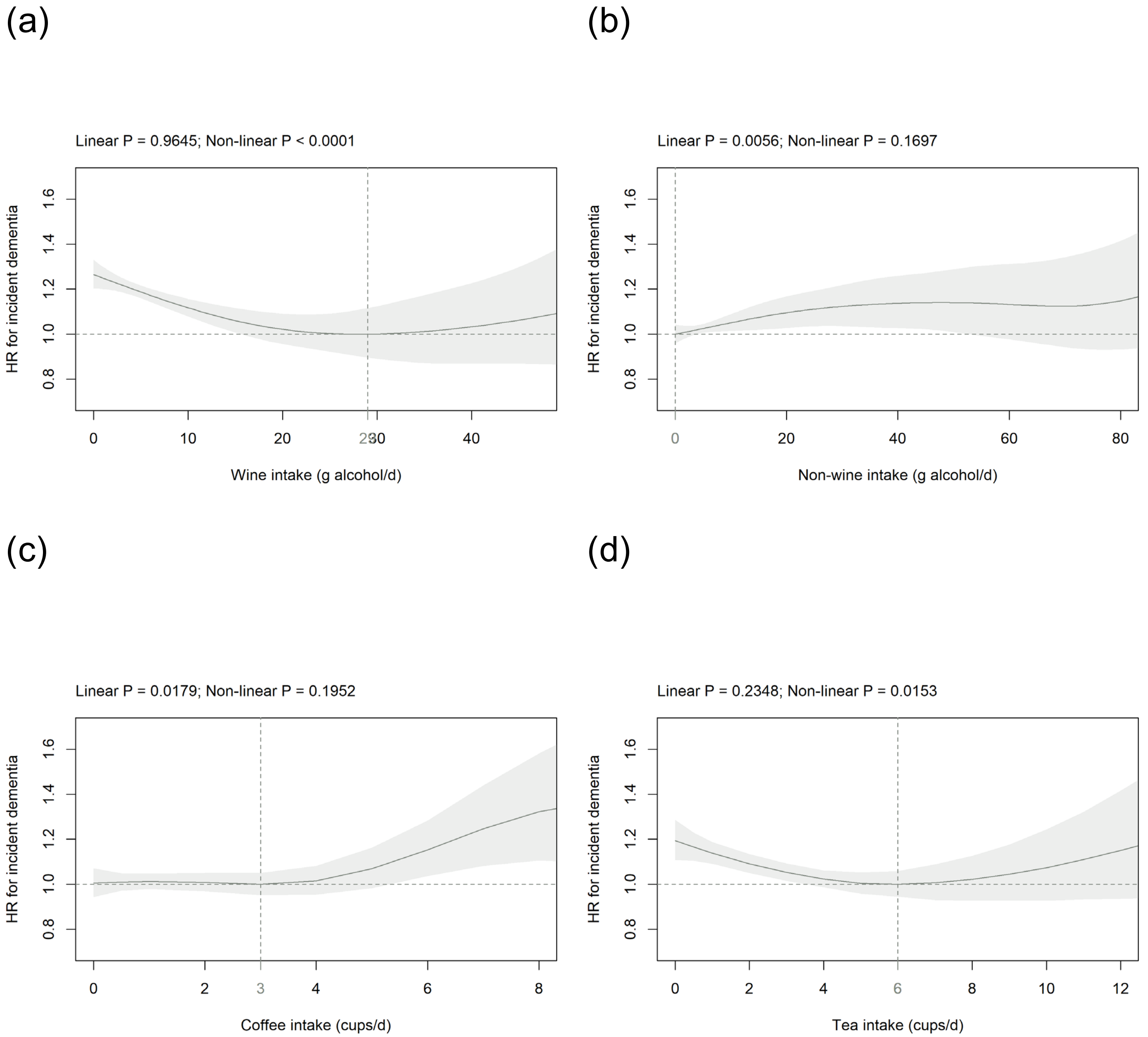

Figure S3. Association of (a) wine intake (g alcohol/d), (b) non-wine intake (g alcohol/d), (c) coffee intake (cups/d), and (d) tea intake (cups/d) with dementia risk in all participants after removal of ICD code G31. Data are adjusted and presented as indicated in Supplementary Figure S2.

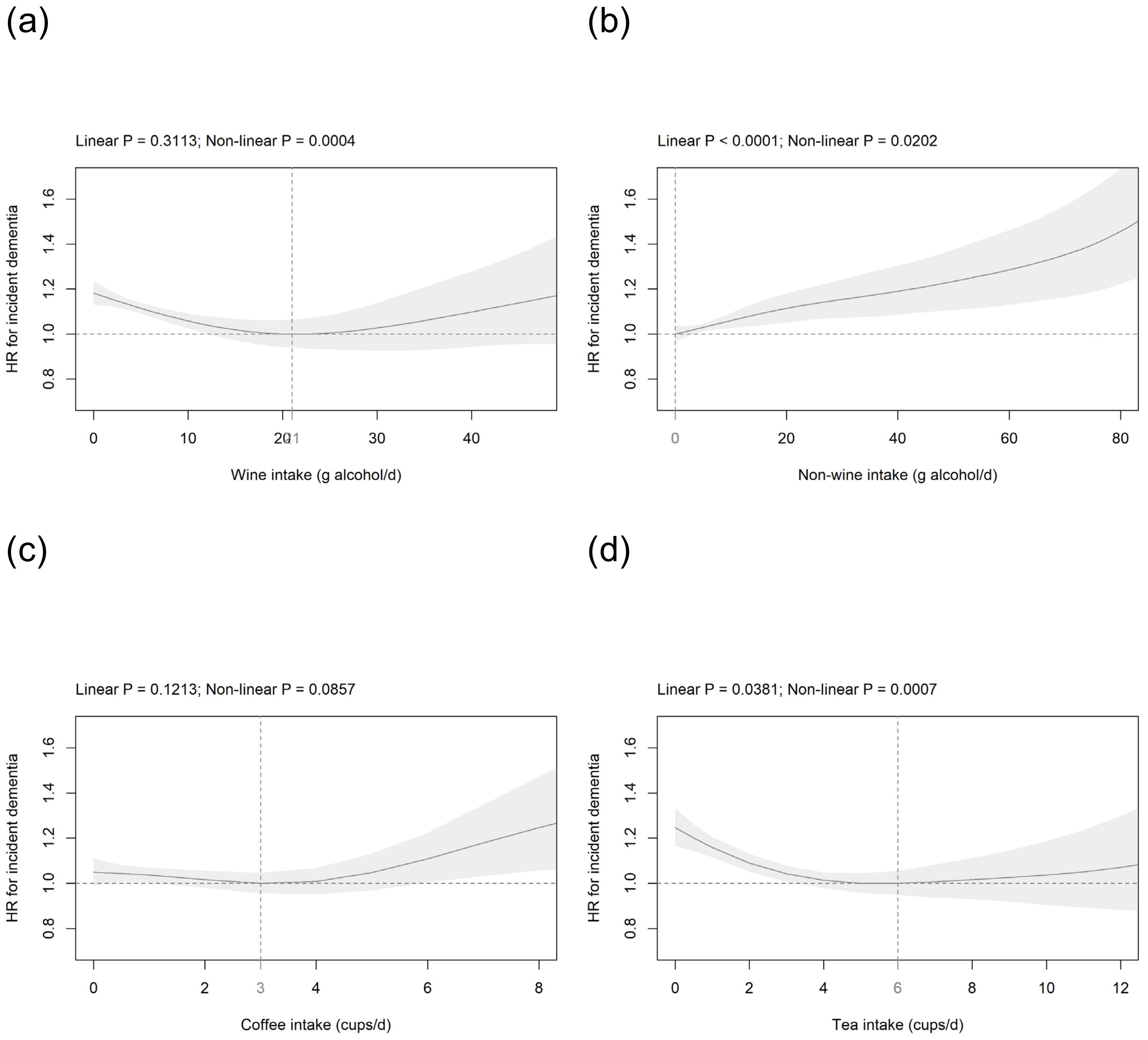

Figure S4. Association of (a) wine intake (g alcohol/d), (b) non-wine intake (g alcohol/d), (c) coffee intake (cups/d), and (d) tea intake (cups/d) with dementia risk in all participants with age and percentage body fat included as continuous instead of categorical variables. Data are adjusted and presented as indicated in Supplementary Figure S2.

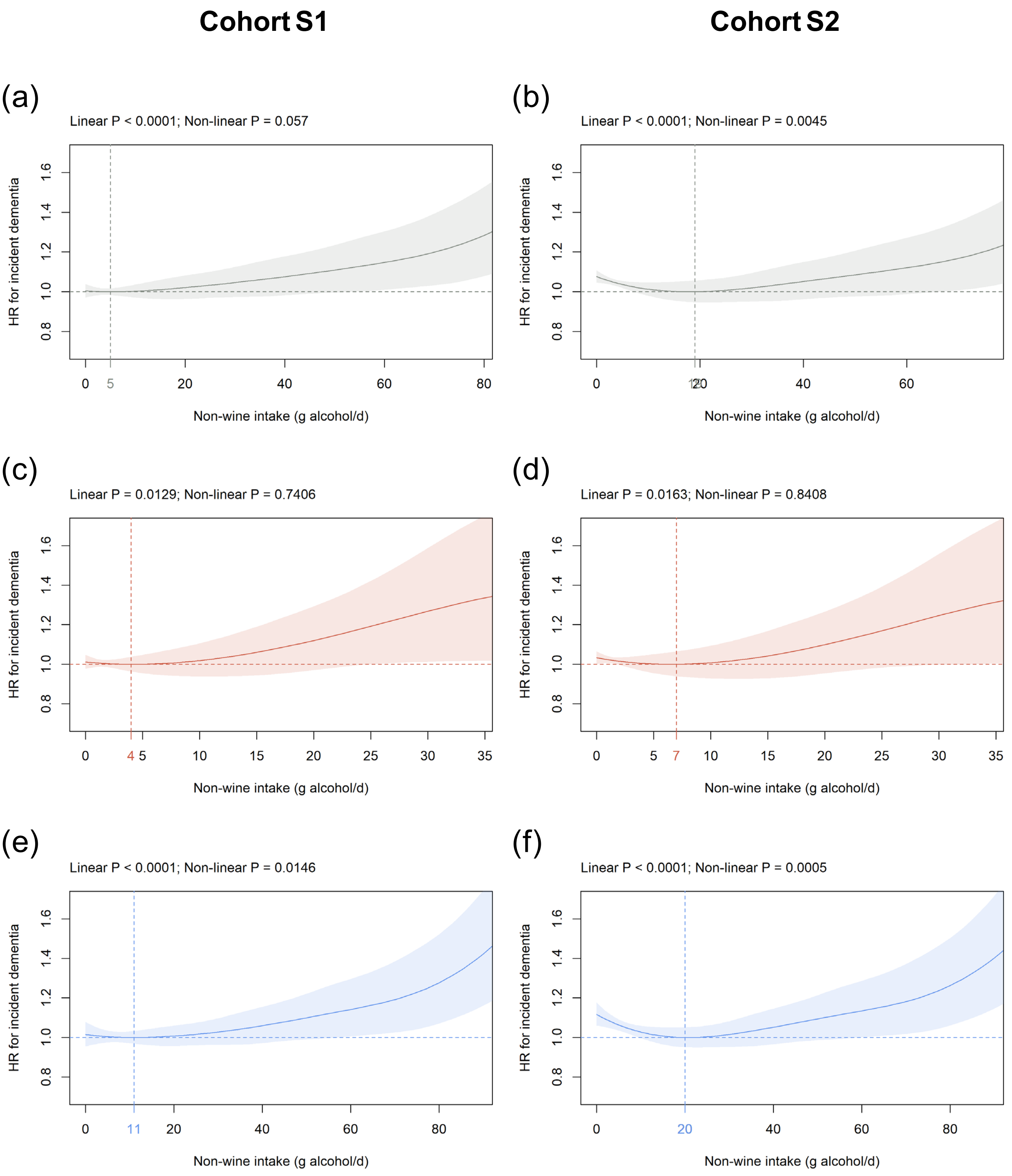

Figure S5. Association of non-wine intake (g alcohol/d) in (a), (b) the total cohort, (c), (d) females, and (e), (f) males with dementia risk in (a), (c), (e) cohort S1 and (b), (d), (f) cohort S2. Data are adjusted and presented as indicated in Supplementary Figure S2.
